# Isolated THATCH domain of End4 is unable to bind F-actin independently in the fission yeast *Schizosaccharomyces pombe*

**DOI:** 10.17912/micropub.biology.000508

**Published:** 2022-01-06

**Authors:** Yuan Ren, Julien Berro

**Affiliations:** 1 Department of Molecular Biophysics and Biochemistry, Yale University; 2 Nanobiology Institute, Yale University; 3 Department of Cell Biology, Yale University School of Medicine

## Abstract

Clathrin mediated endocytosis (CME) in the fission yeast *Schizosaccharomyces pombe* critically depends on the connection between the lipid membrane and F-actin. The fission yeast endocytic protein End4 (homologous to Sla2 in budding yeast and HIP1R in human) contains a N-terminal domain that binds to PIP2 on the membrane, and a C-terminal THATCH domain that is postulated to be a binding partner of F-actin *in vivo*. Purified THATCH domain of the budding yeast Sla2, however, shows low affinity to F-actin* in vitro*. We tested if isolated THATCH domain still has low affinity to F-actin* in vivo*, using TEV protease (TEVp)-mediated protein cleaving to separate the THATCH domain from the rest of End4. Our results indicate that the isolated THATCH domain of End4 is unable to bind F-actin independently *in vivo*, consistent with the low affinity of the THATCH domain to F-actin measured from *in vitro* binding assays.

**Figure 1. Localization of proteins in fission yeast before and after TEVp cleavage in vivo:**
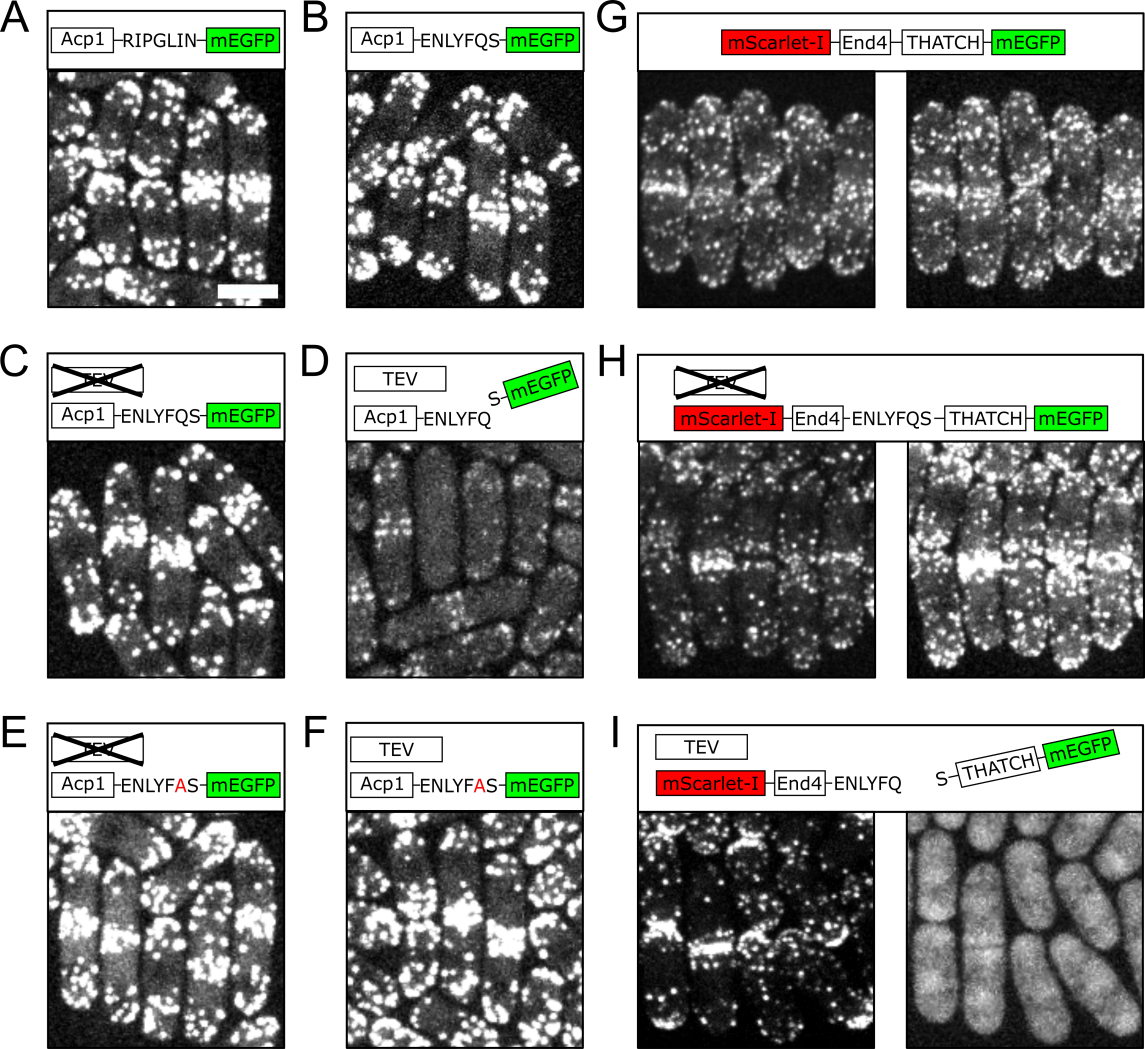
**A:** mEGFP was tagged to the C-terminus of Acp1 with a seven amino acid linker (RIPGLIN). Most of the mEGFP signal is located at endocytic patches. **B:** mEGFP was tagged to the C-terminus of Acp1 with a TEVp recognition peptide (ENLYFQS). The mEGFP signal is localized mostly at endocytic patches similar to wild type in (**A**). **C**–**D:** mEGFP was tagged to the C-terminus of Acp1 with a TEVp recognition peptide, and the expression of TEVp was either inhibited (**C**) or induced (**D**). The expression of TEVp reduced the brightness of the mEGFP signal at endocytic patches in (**D**), suggesting successful cleavage of the mEGFP tag. **E**–**F:** mEGFP was tagged to the C-terminus of Acp1 with a mutated TEVp recognition peptide that cannot be cleaved (ENLYF**A**S), and the expression of TEVp was either inhibited (**E**) or induced (**F**). **G:** End4 was tagged at the N-terminus with mScarlet-I, and at the C-terminus with mEGFP. mScarlet-I (left panel) and mEGFP (right panel) signals co-localize at endocytic patches. **H**–**I:** End4 was tagged at the N-terminus with mScarlet-I, and at the C-terminus with mEGFP. A TEVp recognition peptide was inserted before the THATCH domain, and the expression of TEVp was either inhibited (**H**) or induced (**I**). After TEVp cleavage in (**I**), the THATCH domain of End4 is diffusive in the cytoplasm and the nucleus.

## Description

End4 is an endocytic protein that mediates the connection between the lipid membrane and the actin cytoskeleton in fission yeast (Gottfried, Ehrlich, and Ashery 2010, 4; Iwaki *et al.* 2004, 4; Lacy *et al.* 2018, 4; Engqvist-Goldstein *et al.* 2001; Skruzny *et al.* 2012). The N-terminus of End4 binds to PIP2 on the membrane together with Ent1p, and the C-terminus of End4 shares sequence similarities to the actin binding domain of talin, and is therefore named THATCH (**t**alin-**H**IP1/R/Sla2p **a**ctin-**t**ethering **C**-terminal **h**omology) (Legendre-Guillemin 2004, 4; Baggett, D’Aquino, and Wendland 2003, 4; McCann and Craig 1997; Brett *et al.* 2006). The crystal structures of THATCH domains from HIP1R and talin are five-helix-bundles (Brett *et al.* 2006; Gingras *et al.* 2008). The THATCH domain of Sla2 is critical for linking F-actin to the endocytic coat during CME in budding yeast (Baggett, D’Aquino, and Wendland 2003, 2; Brett *et al.* 2006; Abella *et al.* 2021). The affinity of purified THATCH domains for actin filaments has been measured *in vitro* by multiple groups (Brett *et al.* 2006; Gingras *et al.* 2008; Senetar, Foster, and McCann 2004). Although structurally similar, the THATCH domain from HIP1R has the lowest affinity to actin among all tested THATCH domains, in some cases not significantly higher than that of the negative control with purified GST (Senetar, Foster, and McCann 2004). This discrepancy in the actin-binding ability of the THATCH domain from *in vitro* and *in vivo* data could be explained by cryptic actin-binding sites that are activated *in vivo*, by a) post-translational modifications, b) the binding of other molecules, or c) force transmitted through the rest of the End4 molecule. All three binding site activation mechanisms have been found on talin, the molecule responsible for connecting the membrane to F-actin during cell adhesion through its 13 rod domains including the THATCH domain (Goult, Yan, and Schwartz 2018; Goult, Brown, and Schwartz 2021). A clear way to distinguish mechanism c) from a) and b) is through *in vivo* protein cleaving. By isolating the THATCH domain of End4 from the rest of End4 in the cytoplasm of the fission yeast, the native environment for possible post-translational modifications or binding partners of THATCH are preserved, but force transmission to the THATCH domain is prevented. Consequently, the cellular localization of the isolated THATCH domain is indicative of its direct affinity to F-actin *in vivo*.

We chose to use TEVp to perform *in vivo* protein cleaving, because TEVp has good specificity and only needs seven amino acids as the recognition sequence (Sanchez and Ting 2020). TEVp has been shown to work in diverse situations both *in vivo* and *in vitro* (Harder *et al.* 2008; Raran-Kurussi *et al.* 2017). The coding sequence of TEVp was integrated into the genome of fission yeast by CRISPR/Cas9(Fernandez and Berro 2016), and put under the control of the inducible promoter nmt1 (Forsburg 1993). We first verified that our approach for *in vivo* protein cleaving worked and did not have undesirable effects, by using TEVp to separate the mEGFP tag from a control protein. We used the Acp1 subunit of the canonical actin filament capping protein, which localizes to endocytic sites (Sun *et al.* 2019; Nakano and Mabuchi 2006; Berro and Pollard 2014) and has comparable cytoplasmic concentration to End4 in the fission yeast (Sirotkin *et al.* 2010). When Acp1 was tagged either with a regular linker or the TEVp recognition peptide, the majority of the mEGFP signal was detected at endocytic patches, which were enriched at the poles and the division plane of the fission yeast cells (Fig. 1A-C). These endocytic patches contained a large amount of F-actin(Lacy *et al.* 2018). When the expression of TEVp was induced, we saw a drastic decrease of mEGFP signal from endocytic patches, suggesting the removal of the mEGFP tag from Acp1 molecules (Fig. 1D). As expected, the cleavage of TEVp demonstrated good specificity, and a point mutation in the TEVp recognition peptide prevented protein cleaving (Fig. 1E, 1F).

We then inserted the TEVp recognition peptide prior to the THATCH domain of End4. In wild-type cells, End4 localizes to endocytic patches in a pattern similar to Acp1 (Fig. 1G). The insertion of the TEVp recognition peptide did not change the localization of End4 when TEVp was not expressed (Fig. 1H). When TEVp was expressed, however, the THATCH domain was no longer found at endocytic patches but became diffusive in the cytoplasm and the nucleus, while the N-terminal fragment of End4 still showed polarized localization (Fig. 1I). Failure of the isolated THACTH domain to localize to endocytic patches demonstrates that the affinity of the THATCH domain to F-actin is low *in vivo,* similarly to what was shown *in vitro*. In addition, our results demonstrate that the connection between the THATCH domain and the rest of the End4 molecule is necessary for its binding to actin filaments. We speculate that force transmission through the whole End4 protein is probably involved in exposing the cryptic F-actin binding sites.

## Methods

Yeast strains and media

The *S. pombe* strains used in this study were made though CRISPR/Cas9 mediated genome editing as reported in (Fernandez and Berro 2016), and the edited gene sequences were confirmed by colony PCR and sequencing. The coding sequence of TEVp was inserted into the low complex region 0.5kb upstream of pil1 locus (chromosome III, insertion after 348203) (V. Wood *et al.* 2002; Valerie Wood *et al.* 2012), under the control of the full strength nmt1 promoter (Forsburg 1993). *S. pombe* cells were cultured in YE5S (Yeast Extract supplemented with 0.225 g/L of uracil, lysine, histidine, adenine and leucine), and imaged in EMM5S (Edinburgh Minimum media supplemented with 0.225 g/L of uracil, histidine, adenine, lysine, and leucine) after washing with EMM5S. Yeast cells were cultured at 32 °C with 200rpm shaking overnight to reach OD_595nm_ reading between 0.3 and 0.5. The expression of TEVp was inhibited when yeast cells were cultured in YE5S media. The expression of TEVp was induced by transferring yeast cells into EMM5S media after multiple washes, and cells were imaged after overnight culture in EMM5S to allow for enough time for TEVp mediated protein cleavage.

Microscopy

Cells were imaged at room temperature on gelatin pads (25%) on glass slides. Samples were mounted on a Nikon TiE inverted microscope (Nikon, Tokyo, Japan) with a CSU-W1 Confocal Scanning Unit (Yokogawa Electric Corporation, Tokyo, Japan) with a CFI Plan Apo 100X/1.45NA Phase objective (Nikon, Tokyo, Japan). Images were captured with an iXon Ultra888 EMCCD camera (Andor, Belfast, UK). mEGFP tagged strains were excited with a 488-nm argon-ion laser and filtered with a single band pass filter 510/25. mScarlet-I tagged strains were excited with a 561-nm argon-ion laser and filtered with a single band pass filter 575/25. Fluorescent signals from the whole cell were collected by taking 21 optical sections each with 0.5µm thickness, and 2D maximum projected images were created for each strain by the Fiji distribution of ImageJ (Schindelin *et al.* 2012). All Acp1 strains were imaged and displayed with the same settings. For End4 strains, the mEGFP channel was imaged and displayed with the same settings, and the mScarlet-I channel was imaged and displayed with the same settings.

## Reagents

**Table:** 

Name	Genotype	Used in	Source
SpJB366	acp1-mEGFP fex1Δ fex2Δ ade6-M216 his3-D1 leu1-32 ura4-D18 h-	A	This Study
SpJB524	acp1-TEVSite-mEGFP fex1Δ fex2Δ ade6-M216 his3-D1 leu1-32 ura4-D18 h-	B	This Study
SpJB535	nmt-TEVp acp1-TEVSite-mEGFP fex1Δ fex2Δ ade6-M216 his3-D1 leu1-32 ura4-D18 h-	C, D	This Study
SpJB561	mScarlet-I-end4-mEGFP fex1Δ fex2Δ ade6-M216 his3-D1 leu1-32 ura4-D18 h-	G	(Ren *et al.* 2021)
SpJB572	nmt-TEVp mScarlet-I-end4-TEVSite-end4-mEGFP fex1Δ fex2Δ ade6-M216 his3-D1 leu1-32 ura4-D18 h-	H, I	This Study
SpJB583	nmt-TEVp acp1-TEVSite_QP1A-mEGFP fex1Δ fex2Δ ade6-M216 his3-D1 leu1-32 ura4-D18 h-	E, F	This Study
